# Cardiovascular magnetic resonance imaging-derived intraventricular pressure gradients in ST-segment elevation myocardial infarction: a long-term follow-up study

**DOI:** 10.1093/ehjimp/qyae009

**Published:** 2024-02-09

**Authors:** Lara S F Konijnenberg, Casper W H Beijnink, Maarten van Lieshout, Jacqueline L Vos, Laura Rodwell, Vicente Bodi, José T Ortiz-Pérez, Niels van Royen, José Rodriguez Palomares, Robin Nijveldt

**Affiliations:** Department of Cardiology, Radboud University Medical Center, Geert Grooteplein Zuid 10, 6525 GA Nijmegen, The Netherlands; Department of Cardiology, Radboud University Medical Center, Geert Grooteplein Zuid 10, 6525 GA Nijmegen, The Netherlands; Department of Cardiology, Radboud University Medical Center, Geert Grooteplein Zuid 10, 6525 GA Nijmegen, The Netherlands; Department of Cardiology, Radboud University Medical Center, Geert Grooteplein Zuid 10, 6525 GA Nijmegen, The Netherlands; Department of Epidemiology and Biostatistics, Radboud University Medical Center, 6525 GA Nijmegen, The Netherlands; Department of Cardiology, Hospital Clínico Universitario de Valencia, 46010 Valencia, Spain; Department of Medicine, Faculty of Medicine and Odontology, University of Valencia, 46010 Valencia, Spain; Instituto de Investigación Sanitaria (INCLIVA), 46010 Valencia, Spain; Centro de Investigación Biomédica en Red de Enfermedades Cardiovasculares (CIBERCV), 28022 Madrid, Spain; Department of Cardiology, Institut d'Investigacions Biomèdiques August Pi I Sunyer (IDIBAPS), 08036 Barcelona, Spain; Clínic Cardiovascular Institute, Hospital Clinic, Universitat de Barcelona, 08036 Barcelona, Spain; Department of Cardiology, Radboud University Medical Center, Geert Grooteplein Zuid 10, 6525 GA Nijmegen, The Netherlands; Centro de Investigación Biomédica en Red de Enfermedades Cardiovasculares (CIBERCV), 28022 Madrid, Spain; Department of Cardiology, Hospital Universitario Vall d'Hebron, Institut de Recerca, Universitat Autònoma de Barcelona, 08035 Barcelona, Spain; Department of Cardiology, Radboud University Medical Center, Geert Grooteplein Zuid 10, 6525 GA Nijmegen, The Netherlands

**Keywords:** ST-elevation myocardial infarction, cardiac magnetic resonance imaging, microvascular injury, feature tracking, intraventricular pressure gradients

## Abstract

**Aims:**

Recently, novel post-processing tools have become available that measure intraventricular pressure gradients (IVPGs) on routinely obtained long-axis cine cardiac magnetic resonance (CMR) images. IVPGs provide a comprehensive overview of both systolic and diastolic left ventricular (LV) functions. Whether IVPGs are associated with clinical outcome after ST-elevation myocardial infarction (STEMI) is currently unknown. Here, we investigated the association between CMR-derived LV-IVPGs and major adverse cardiovascular events (MACE) in a large reperfused STEMI cohort with long-term outcome.

**Methods and results:**

In this prospectively enrolled multi-centre cohort study, 307 patients underwent CMR within 14 days after the first STEMI. LV-IVPGs (from apex-to-base) were estimated on the long-axis cine images. During a median follow-up of 9.7 (5.9–12.5) years, MACE (i.e. composite of cardiovascular death and *de novo* heart failure hospitalisation) occurred in 49 patients (16.0%). These patients had larger infarcts, more often microvascular injury, and impaired LV-IVPGs. In univariable Cox regression, overall LV-IVPG was significantly associated with MACE and remained significantly associated after adjustment for common clinical risk factors (hazard ratio (HR) 0.873, 95% confidence interval (CI) 0.794–0.961, *P* = 0.005) and myocardial injury parameters (HR 0.906, 95% CI 0.825–0.995, *P* = 0.038). However, adjusted for LV ejection fraction and LV global longitudinal strain (GLS), overall LV-IVPG does not provide additional prognostic information (HR 0.959, 95% CI 0.866–1.063, *P* = 0.426).

**Conclusion:**

Early after STEMI, CMR-derived LV-IVPGs are univariably associated with MACE and this association remains significant after adjustment for common clinical risk factors and measures of infarct severity. However, LV-IVPGs do not add prognostic value to LV ejection fraction and LV GLS.

## Graphical Abstract

**Figure:**
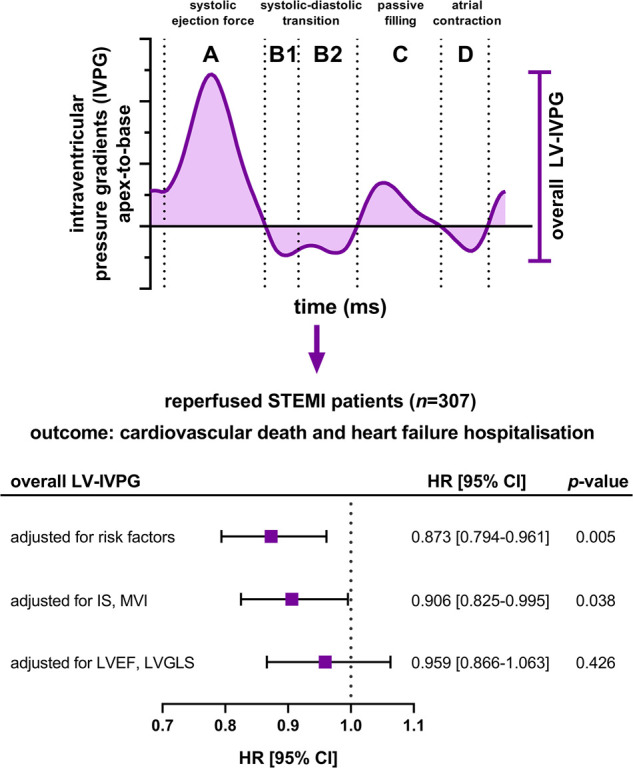

## Introduction

ST-elevation myocardial infarction (STEMI) can lead to geometrical changes and subsequent remodelling of the myocardium. Although remodelling is initially mainly adaptive, sustained remodelling can progress to structural alterations and left ventricular (LV) dysfunction.^1,2^ Adverse LV remodelling is observed in roughly 25% of STEMI patients^3^ and is associated with poor clinical outcome.^4,5^ Several parameters are known to attribute to adverse LV remodelling, including baseline LV ejection fraction (LV EF),^6^ infarct size,^7^ and the presence of microvascular injury (MVI) in the infarct core.^8^ However, the exact underlying mechanism of adverse LV remodelling remains poorly understood and therefore also poses challenges in timely identifying patients at higher risk for unfavourable outcome after STEMI. Exploring LV haemodynamics early post-STEMI could help to better explain the transition to adverse LV remodelling and may thereby help to identify patients at high risk for worse clinical outcome.

Cardiac magnetic resonance (CMR) imaging allows the assessment of both morphological and functional changes post-STEMI.^9^ LV function can be assessed by volumetric parameters (e.g. LV EF) and myocardial deformation parameters (e.g. LV global longitudinal strain (GLS)). Recently, it has become possible to assess haemodynamic forces (HDF) on routinely obtained long-axis steady-state free precession cine images.^10,11^ HDF analysis is based on the estimation of intraventricular pressure gradients (IVPGs). Essentially, IVPGs are generated by the force exchanged between blood and its surrounding myocardium during the cardiac cycle and drive intracardiac blood flow. In contrast to LV EF and LV GLS, assessment of IVPGs provides a comprehensive overview of both systolic and diastolic LV functions^11^ and may assist in the detection of early stage systolic and/or diastolic LV dysfunction post-STEMI. It is assumed that disturbed IVPGs initiate adverse LV remodelling^12,13^ and precede alterations observed with conventional LV function imaging parameters.^14^ Recently, the role of IVPGs in adverse LV remodelling post-STEMI was investigated, showing that diastolic IVPG misalignment 1 week after STEMI was independently associated with adverse LV remodelling at 4 months follow-up.^15^ However, the association between LV-IVPG and major adverse cardiovascular events (MACE) in STEMI is so far unexplored and hence is the focus of this study. Identification of potential early predictors of adverse events may improve patient risk stratification, tailor surveillance, and eventually may improve clinical outcome.

In the present study, we investigated whether CMR-derived LV-IVPGs were associated with cardiovascular death and *de novo* heart failure hospitalisation in a large reperfused STEMI cohort with long-term follow-up.

## Methods

### Study design and population

The study was conducted in accordance with the Declaration of Helsinki and approved by the Medical Ethics Review Committee of the participating centres. All patients provided written informed consent prior to study participation. This cohort study included patients from a large multi-centre STEMI CMR registry enrolled between December 2001 and December 2016 in Vall d’Hebron University Hospital Barcelona and Valencia University Hospital, of which the results have been published previously.^16,17^ For this cohort study, the main inclusion criteria were patients diagnosed with the first STEMI, CMR examination within 14 days after STEMI, and complete follow-up data. The main exclusion criteria were haemodynamically instability, incomplete or poor quality CMR, severe valvular disease, atrial fibrillation, or any other relevant cardiac pathological characteristics.

### Demographic and clinical characteristics

Demographic and clinical characteristics were identified from the patients’ medical records. Patients were treated according to international clinical guidelines.^18^ Multi-vessel disease was defined as the presence of a non-culprit artery stenosis>70% on coronary angiography.

### CMR acquisition

All patients underwent a comprehensive CMR scan (1.5 or 3.0 T clinical MRI scanner, Magnetom Symphony, Sonata Magnetom or Somaton Trio, Siemens) with standard two-chamber, three-chamber, and four-chamber long-axis and short-axis views. Cardiac function was assessed using steady-state free precession cine imaging (typical parameters: slice thickness 6 mm, repetition time 2.8 ms, echo time 1.2 ms, flip angle 58°, and temporal resolution <50 ms). Subsequently, the late gadolinium-enhanced (LGE) images were acquired 10–12 min after administration of a gadolinium-based contrast agent (0.1–0.2 mmol/kg) using a 2D segmented inversion recovery gradient-echo sequence (typical parameters: slice thickness 6 mm, voxel size 2.0 × 1.6 mm^2^) using the same slice position as the cine images. Inversion time was adjusted to null the signal of the viable myocardium.

#### CMR global LV function analysis

Briefly, consecutive short-axis cine images were manually traced to calculate LV mass, volumes, and LV EF (Medis Medical Imaging BV, Medis Suite version 3.2.60.6). The infarct area was defined as the myocardial area with hyper-enhancement on the LGE images using manual planimetry. Infarct size was expressed as a percentage of LV mass (% LVM). MVI, a measure of more severe myocardial injury with haemorrhage and microvascular obstruction, was visually defined as a contrast-devoid infarct core within a contrast-enhanced infarct area on LGE imaging. MVI was expressed as present or absent.

#### CMR feature tracking analysis

Feature tracking analysis was performed on the long-axis cine images in two-, three-, and four-chamber to assess LV GLS (Medis Medical Imaging BV, Medis Suite version 3.2.60.6 and QStrain version 3.2.4.2). LV endocardial contours were manually drawn in the end-systolic and end-diastolic phases. Subsequently, contours were automatically tracked in consecutive frames and manually adjusted when necessary. The software calculates the myocardial shortening throughout the cardiac cycle and provides LV GLS.

#### CMR LV HDF analysis

CMR-derived LV HDF analysis is based on the estimation of IVPGs during the cardiac cycle. IVPGs are determined using a formula based on the Navier–Stokes equation that assesses the myocardial movement and velocity, and the blood velocity over the valves (more details can be found in Pedrizzetti,^10^ Vallelonga *et al*.,^11^ and Vos *et al*.^19^). Briefly, consecutive long-axis endocardial contours were obtained using feature tracking analysis, resulting in a 3D endocardial border movement reconstruction. The diameter of the aortic valve was manually drawn in peak-systole on three-chamber long-axis and the mitral valve in early diastole on two-, three-, and four-chamber long-axis planes. Subsequently, the outflow area was calculated. The longitudinal (i.e. apex-to-base) and lateral–septal IVPG time curve was generated by the software and represents the variation of IVPGs throughout the cardiac cycle. Under physiological conditions, IVPGs are predominantly aligned with the LV longitudinal axis.^20^ IVPGs (in Newton) are normalised to their corresponding LV volume and subsequently to the gravity of blood (in Newton), resulting in a dimensionless parameter. The overall amplitude of the longitudinal force and the lateral–septal force during the entire cardiac cycle is represented by the area under the curve. The ratio between lateral–septal and longitudinal force (in %) was used to assess the relative distribution of IVPGs in the LV. Using the IVPG time curve and the volume curve, five phases can be distinguished (see Supplementary data online, *Figure S1*): A, B (B1 and B2), C, and D. The positive vector ***A-wave*** represents the systolic ejection phase. The negative vector ***B-wave*** represents the systolic–diastolic transition, in which ***B1*** includes the end-systolic LV contraction slow-down phase and ***B2*** the diastolic suction. When LV apical pressure temporarily exceeds the LV basal pressure, reversal of IVPG at the systolic–diastolic transition can be observed. The positive vector E-wave deceleration ***C*** represents the diastolic filling slow-down phase and ends when the pressure between the left atrium and LV equals. Lastly, the negative vector A-wave acceleration ***D*** represents the atrial contraction in active, late diastole.

### Clinical endpoints

The primary endpoint was the occurrence of MACE, defined as a composite of cardiovascular death and *de novo* heart failure hospitalisation. Heart failure hospitalisation was defined as admission for >24 h with a primary diagnosis of heart failure or progressive deterioration of heart failure requiring intensified treatment. Follow-up data of patients from Vall d’Hebron University Hospital Barcelona were retrieved from local chart review and/or telephone interviews, whereas the data of patients from Valencia University Hospital were retrieved from local electronic patient records.

### Statistical analysis

Categorical data were presented as frequencies and corresponding percentages. Continuous data were presented as mean ± standard deviation for normally distributed variables or median with interquartile range (IQR) for non-normally distributed variables. Categorical data were compared using a χ^2^ test or Fisher’s exact test. Continuous data were compared using an independent-sample *T*-test or Mann–Whitney *U* test where appropriate. Correlations between variables were assessed using Pearson’s *R*. To identify candidate prognostic factors for MACE (i.e. the composite of cardiovascular death and *de novo* heart failure hospitalisation), univariable and multi-variable Cox proportional hazard regression analyses were performed, based on time to first event. All numeric variables were included as continuous variables. Non-cardiovascular death was censored and interpreted as a cause-specific hazard.^21^ To prevent overfitting in multi-variable Cox regression, no more than five variables were included. With respect to the number of events, three multi-variable Cox regression models were created. Model A adjusted for clinical determinants for MACE (i.e. age, sex, diabetes mellitus, hypertension, smoking). Model B adjusted for markers of myocardial injury (i.e. infarct size, presence of MVI). Model C adjusted for conventional LV function parameters (i.e. LV EF, LV GLS). For model C, multi-collinearity was assessed by the variance inflation factor (VIF). VIF > 5 was interpreted as relevant collinearity and >10 as serious collinearity.^22^ For each model, cause-specific hazard ratios (HRs) and 95% confidence intervals (95% CIs) were reported. A *P*-value <0.05 was considered statistically significant. All statistical analyses were performed using SPSS statistics (IBM SPSS Statistics 27, Chicago, IL, USA).

## Results

### Study population

In total, 361 STEMI patients were screened. After exclusion due to incomplete or insufficient quality CMR, atrial fibrillation, or other severe cardiac pathology, 307 patients were included for analysis (see Supplementary data online, *Figure S2*). Male sex predominated (*n* = 264, 86.0%), mean age was 58.2 ± 11.5 years, and 298 patients (97.1%) were revascularised with PCI. Multi-vessel disease was observed in 78 patients (25.4%). The median time to reperfusion was 180.0 (139.0–260.8) min and the follow-up duration was 9.7 (5.9–12.5) years. Patients received evidence-based concomitant medical therapy at hospital discharge (*Table 1*).

**Table 1:** Baseline characteristics

Total study population (*n* = 307)	
Age (years)	58.2 ± 11.5
Male, *n* (%)	264 (86.0)
Body surface area (m^2^)	1.9 ± 0.2
Follow-up duration (years)	9.7 (5.9–12.5)
Medical history, *n* (%)	
Diabetes mellitus	46 (15.0)
Family history of CAD	27 (8.8)
Hypercholesterolaemia	112 (36.5)
Hypertension	136 (44.3)
Smoking, ‘current or in the past’	197 (64.2)
Reperfusion therapy, *n* (%)	
Primary PCI	208 (67.8)
Facilitated PCI	70 (22.8)
Thrombolytics	8 (2.6)
Rescue PCI	2 (0.7)
PCI > 12 h	18 (5.9)
CABG	1 (0.3)
Coronary angiography	
Time to reperfusion (min)	180.0 (139.0–260.8)
Culprit artery, *n* (%)	
Left anterior descending coronary artery	180 (58.6)
Left circumflex coronary artery	24 (7.8)
Right coronary artery	103 (33.6)
Multi-vessel disease, *n* (%)	78 (25.4)
Medication at hospital discharge, *n* (%)	
Dual antiplatelet therapy	307 (100)
ACE inhibitor or ARB	229 (74.6)
β-Blocker	250 (81.4)
Mineralocorticoid receptor antagonists	16 (5.2)
Statin	285 (92.8)

Values are presented as mean ± standard deviation, median (IQR), or absolute number (%).

ACE inhibitor, angiotensin-converting enzyme inhibitors; ARB, angiotensin II receptor blockers; CABG, coronary artery bypass grafting; CAD, coronary artery disease; PCI, percutaneous coronary intervention.

### CMR characteristics

CMR scans were performed 6 (4–8) days after STEMI. All CMR parameters are described in *Table 2*. The median infarct size was 20.1 (11.2–29.5) of LVM, 179 patients (58.3%) had an anterior wall infarct, and 140 patients (45.6%) showed MVI on LGE CMR. Regarding LV function, the mean LV EF and LV GLS were 50.5 ± 11.1 and −13.1 ± 4.7%, respectively. LV HDF analysis showed an overall LV-IVPG (i.e. longitudinal force) of 10.7 ± 3.6, lateral–septal force of 2.8 ± 0.9, systolic ejection ‘A’ of 18.1 ± 7.4 and E-wave deceleration ‘C’ of 6.7 ± 3.3. Both overall LV-IVPG and systolic ejection ‘A’ were significantly correlated to LV EF (*R* = 0.589, *P* < 0.001; *R* = 0.563, *P* < 0.001, respectively) and LV GLS (*R* = −0.408, *P* < 0.001; *R* = −0.349, *P* < 0.001, respectively).

**Table 2:** CMR left ventricular parameters

**Global left ventricular parameters**	
LV EDV (mL)	149.0 ± 39.2
LV indexed EDV (mL/m^2^)	78.3 ± 20.3
LV ESV (mL)	75.0 ± 30.9
LV indexed ESV (mL/m^2^)	39.5 ± 16.7
LV EF (%)	50.5 ± 11.1
SV (mL)	74.1 ± 21.8
SV indexed (mL/m^2^)	38.7 ± 10.5
LV ED mass (g)	133.4 ± 39.6
LV indexed ED mass (g/m^2^)	69.8 ± 19.4
LV GLS (%)	−13.1 ± 4.7
Infarct size (g)	24.0 (14.3–40.1)
Infarct size (% LVM)	20.1 (11.2–29.5)
Anterior infarct location, *n* (%)	179 (58.3)
MVI, *n* (%)	140 (45.6)
**Left ventricular IVPGs**	
Overall LV-IVPG (i.e. longitudinal force)	10.7 ± 3.6
Lateral–septal force	2.8 ± 0.9
Ratio lateral–septal/longitudinal force (%)	26.7 ± 7.2
Systolic ejection ‘A’	18.1 ± 7.4
Peak systolic force ‘A-peak’	28.6 ± 11.5
Systolic–diastolic transition ‘B’	−6.3 ± 2.5
Systolic slowdown ‘B1’	−5.2 ± 2.8
Diastolic suction ‘B2’	−7.2 ± 3.4
B-wave reversal, *n* %	84 (27.4)
E-wave deceleration ‘C’	6.7 ± 3.3
A-wave acceleration ‘D’	−3.8 ± 2.2

Values are presented as mean ± standard deviation, median (IQR), or absolute number (%).

CMR, cardiac magnetic resonance imaging; ED, end-diastolic; EDV, end-diastolic volume; EF, ejection fraction; ESV, end-systolic volume; GLS, global longitudinal strain; IVPG, intraventricular pressure gradient; LV, left ventricle; LVM, left ventricular myocardial mass; MVI, microvascular injury; SV, stroke volume.

### Association of LV-IVPGs with clinical outcomes

In this study cohort, 44 patients (14.3%) died of whom 22 (50.0%) were ascribed to cardiovascular death. Cardiovascular death included death from heart failure (*n* = 8, 36.4%), life-threatening arrythmias (*n* = 9, 40.1%), ischaemic heart disease (*n* = 1, 4.5%), and stroke (*n* = 4, 18.2%). In total, 49 patients (16.0%) reached the composite endpoint [i.e. time to first event; *de novo* heart failure hospitalisation (*n* = 34) and cardiovascular death (*n* = 15)] during a median follow-up of 9.7 (5.9–12.5) years. These patients were older and presented with larger infarcts and more often with MVI. In addition, they had worse LV EF and LV GLS values. Based on LV HDF analysis, they had impaired systolic LV-IVPGs and showed more often reversal of LV-IVPG at systolic–diastolic transition. Diastolic LV-IVPGs were comparable between groups (see Supplementary data online, *Table S1*).

In univariable Cox regression analysis, a significant association was found between overall LV-IVPG (HR 0.866, 95% CI 0.790–0.949, *P* = 0.002), systolic force (systolic ejection ‘A’ HR 0.934, 95% CI 0.891–0.978, *P* = 0.004; peak systolic force ‘A-peak’ HR 0.960, 95% CI 0.933–0.988, *P* = 0.006), and diastolic force (E-wave deceleration ‘C’ HR 0.890, 95% CI 0.804–0.984, *P* = 0.024), and MACE. In multi-variable Cox regression analysis, overall LV-IVPG remained significantly associated with MACE after adjustment for clinical determinants for MACE (*Model A,* HR 0.873, 95% CI 0.794–0.961, *P* = 0.005) and parameters of myocardial injury (*Model B,* HR 0.906, 95% CI 0.825–0.995, *P* = 0.038). In a multi-variable LV function model, including overall LV-IVPG, LV EF, and LV GLS (*Model C*, VIF 1.76), only LV GLS remained significantly associated with MACE (HR 1.114, 95% CI 1.023–1.215, *P* = 0.014) (*Table 3*).

**Table 3:** Candidate prognostic factors for cardiovascular death and *de novo* heart failure hospitalisation

*N* = 306*	Univariable HR (95% CI)	*P*-value	Multi-variable HR (95% CI)	*P*-value
Model A, Clinical covariates				
Overall LV-IVPG (i.e. longitudinal force)	0.866 (0.790–0.949)	**0.002**	0.873 (0.794–0.961)	**0.005**
Age, years	1.078 (1.049–1.107)	**<0.001**	1.074 (1.041–1.108)	**<0.001**
Diabetes mellitus, yes	1.268 (0.593–2.711)	0.541		
Hypertension, yes	1.616 (0.915–2.854)	0.098	1.044 (0.560–1.948)	0.891
Sex, male	0.655 (0.317–1.355)	0.254	1.070 (0.486–2.356)	0.866
Smoking, yes	0.417 (0.236–0.738)	**0.003**	0.896 (0.471–1.706)	0.738
Model B, CMR myocardial injury parameters				
Overall LV-IVPG (i.e. longitudinal force)	0.866 (0.790–0.949)	**0.002**	0.906 (0.825–0.995)	**0.038**
Infarct size, %LVM	1.038 (1.020–1.056)	**<0.001**	1.033 (1.011–1.055)	**0.003**
MVI, yes	1.789 (1.007–3.178)	**0.047**	0.952 (0.491–1.846)	0.885
Model C, CMR left ventricular function parameters				
Overall LV-IVPG (i.e. longitudinal force)	0.866 (0.790–0.949)	**0.002**	0.959 (0.866–1.063)	0.426
LV EF, %	0.941 (0.917–0.967)	**<0.001**	0.977 (0.941–1.015)	0.241
LV GLS, %	1.171 (1.096–1.251)	**<0.001**	1.114 (1.023–1.215)	**0.014**

*P*-values <0.05 are depicted in bold.

EF, ejection fraction; HR, hazard ratio; IVPG, intraventricular pressure gradient; LV, left ventricle; LVM, left ventricular myocardial mass; MVI, microvascular injury.

^*^One patient was excluded from the analysis because of a missing overall LV-IVPG.

### Impact of infarct severity on LV-IVPGs

**Figure 1:**
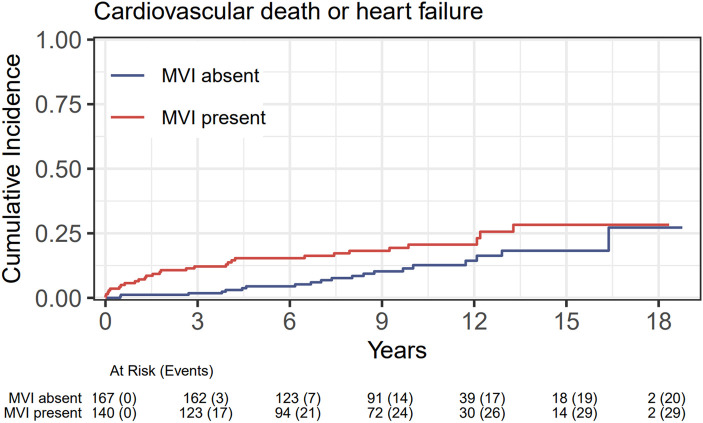
Cumulative incidence of the primary composite endpoint of cardiovascular death and *de novo* heart failure hospitalisation for patients with (red, upper line) and without MVI (blue, lower line). The *y*-axis represents the cumulative incidence. The *x*-axis represents the time in years since STEMI diagnosis.

In an exploratory analysis, we evaluated the impact of the infarct severity on LV HDF. Patients with large infarcts, defined as an infarct size ≥20.1% of LV, showed significantly impaired LV HDF, including all systolic and diastolic LV-IVPGs, compared with patients with smaller infarcts. Additionally, the lateral–septal/longitudinal force ratio was significantly higher, and reversal of LV-IVPG at systolic–diastolic transition was more frequently observed in patients with large infarcts (*Table 4*). Regarding infarct location, no differences in systolic LV-IVPGs were observed between anterior (*n* = 179) and non-anterior infarcts (*n* = 128). In the systolic–diastolic transition phase, the systolic slowdown ‘B1’ was significantly impaired (−4.3 ± 2.4 vs. −6.4 ± 2.7, *P* < 0.001) and reversal of LV-IVPG was more frequently observed (39.1% vs. 10.9%, *P* < 0.001) in anterior infarcts. Furthermore, A-wave acceleration ‘D’ (−3.6 ± 2.1 vs. −4.1 ± 2.2, *P* = 0.036) was significantly impaired in anterior infarcts compared with non-anterior infarcts. Besides infarct size and infarct location, the infarct severity can be assessed by the presence or absence of MVI in the infarct core. Comparable to patients with large infarcts, patients with MVI (*n* = 140) showed significantly impaired systolic and diastolic LV-IVPGs compared with patients without MVI. Reversal of LV-IVPG at systolic–diastolic transition was observed equally often (*Table 5*, *example in* Supplementary data online, *Figure S3*). Related to clinical outcome, MACE was more often observed in patients with large infarcts (21.6 vs. 10.4%, *P* = 0.007), in patients with anterior wall infarcts (20.0 vs. 10.9%, *P* = 0.042), and in patients with MVI (20.7 vs. 12.0%, *P* = 0.037). This significant difference in MACE between groups was mainly driven by *de novo* heart failure hospitalisation (16.3% in large infarcts vs. 5.8%, *P* = 0.003; 14.5% in anterior wall infarcts vs. 6.3%, *P* = 0.023; and 16.4% in patients with MVI vs. 6.6%, *P* = 0.006, *Figure 1*) in the first years after STEMI diagnosis.

**Table 4:** CMR left ventricular parameters in patients with larger compared with smaller infarcts

	Infarct size ≥20.1% (*n* = 153)	Infarct size <20.1% (*n* = 154)	*P*-value
Global left ventricular parameters			
LV EDV (mL)	157.7 ± 39.8	140.3 ± 36.8	<0.001
LV indexed EDV (mL/m^2^)	83.5 ± 21.7	73.0 ± 17.2	<0.001
LV ESV (mL)	88.4 ± 31.2	61.5 ± 24.0	<0.001
LV indexed ESV (mL/m^2^)	47.0 ± 17.3	32.0 ± 12.0	<0.001
LV EF (%)	44.5 ± 9.4	56.6 ± 9.2	<0.001
LV GLS (%)	−10.8 ± 4.2	−15.4 ± 4.0	<0.001
Anterior infarct location, *n* (%)	111 (72.5)	68 (44.2)	<0.001
MVI, *n* (%)	105 (68.6)	35 (22.7)	<0.001
Left ventricular IVPGs			
Overall LV-IVPG (i.e. longitudinal force)	9.6 ± 3.1	11.8 ± 3.8	<0.001
Lateral–septal force	2.6 ± 0.8	2.9 ± 1.0	<0.001
Ratio lateral–septal/longitudinal force (%)	27.7 ± 7.5	25.8 ± 6.8	0.020
Systolic ejection ‘A’	16.2 ± 6.9	20.1 ± 7.3	<0.001
Peak systolic force ‘A-peak’	25.3 ± 10.4	31.9 ± 11.7	<0.001
Systolic–diastolic transition ‘B’	−5.8 ± 2.2	−6.9 ± 2.6	<0.001
Systolic slowdown ‘B1’	−4.5 ± 2.4	−5.8 ± 2.9	<0.001
Diastolic suction ‘B2’	−6.6 ± 3.1	−7.7 ± 3.6	0.006
B-wave reversal, *n* %	50 (32.7)	34 (22.1)	0.037
E-wave deceleration ‘C’	6.2 ± 3.1	7.2 ± 3.4	0.009
A-wave acceleration ‘D’	−3.4 ± 2.1	−4.3 ± 2.2	<0.001

Values are presented as mean ± standard deviation, median (IQR), or absolute number (%), and assessed by independent-samples *T*-test, Mann–Whitney *U* test, or χ^2^ test, respectively.

CMR, cardiac magnetic resonance imaging; EDV, end-diastolic volume; EF, ejection fraction; ESV, end-systolic volume; GLS, global longitudinal strain; IVPG, intraventricular pressure gradient; LV, left ventricle; MVI, microvascular injury.

**Table 5:** CMR left ventricular parameters in patients with and without MVI

	MVI present (*n* = 140)	MVI absent (*n* = 167)	*P*-value
Global left ventricular parameters			
LV EDV (mL)	159.3 ± 41.4	140.4 ± 35.2	<0.001
LV indexed EDV (mL/m^2^)	83.4 ± 22.4	74.0 ± 17.2	<0.001
LV ESV (mL)	88.7 ± 33.4	63.5 ± 23.2	<0.001
LV indexed ESV (mL/m^2^)	46.6 ± 18.4	33.5 ± 12.3	<0.001
LV EF (%)	45.2 ± 9.8	55.1 ± 10.4	<0.001
LV GLS (%)	−11.4 ± 4.7	−14.5 ± 4.2	<0.001
Infarct size (% LVM)	27.5 (19.5–36.9)	13.8 (7.6–21.7)	<0.001
Anterior infarct location, *n* (%)	88 (62.9)	91 (54.5)	0.139
Left ventricular IVPGs			
Overall LV-IVPG (i.e. longitudinal force)	9.7 ± 3.0	11.6 ± 3.9	<0.001
Lateral–septal force	2.6 ± 0.8	2.9 ± 1.0	0.003
Ratio lateral–septal/longitudinal force (%)	27.6 ± 7.5	26.0 ± 6.9	0.056
Systolic ejection ‘A’	16.1 ± 6.3	19.8 ± 7.8	<0.001
Peak systolic force ‘A-peak’	25.6 ± 9.8	31.1 ± 12.3	<0.001
Systolic–diastolic transition ‘B’	−5.8 ± 2.2	−6.8 ± 2.6	<0.001
Systolic slowdown ‘B1’	−4.8 ± 2.6	−5.5 ± 2.8	0.030
Diastolic suction ‘B2’	−6.5 ± 3.0	−7.7 ± 3.7	0.002
B-wave reversal, *n* (%)	38 (27.1)	46 (27.5)	0.937
E-wave deceleration ‘C’	6.2 ± 3.0	7.2 ± 3.4	0.007
A-wave acceleration ‘D’	−3.4 ± 1.9	−4.2 ± 2.3	<0.001

Values are presented as mean ± standard deviation, median (IQR), or absolute number (%), and assessed by independent-samples *T*-test, Mann–Whitney *U* test, or χ^2^ test, respectively.

CMR, cardiac magnetic resonance imaging; EDV, end-diastolic volume; EF, ejection fraction; ESV, end-systolic volume; GLS, global longitudinal strain; IVPG, intraventricular pressure gradient; LV, left ventricle; LVM, left ventricular myocardial mass; MVI, microvascular injury.

## Discussion

The aim of the present study was to explore the association between CMR-derived LV-IVPGs and MACE in reperfused STEMI patients. In univariable Cox regression analysis, we showed that both systolic and diastolic LV-IVPGs were associated with MACE during a median follow-up of 9.7 years. Subsequently, we showed that overall LV-IVPG (i.e. longitudinal force) remained significantly associated with MACE after adjustment for parameters of myocardial injury and clinical determinants for MACE. However, its significant association disappeared when overall LV-IVPG was adjusted for LV EF and LV GLS. Furthermore, in an exploratory analysis, we showed that patients with severe myocardial injury, as manifested by large infarcts or infarcts with MVI, showed significantly smaller HDF amplitudes, including all systolic and diastolic LV-IVPGs, compared with those with less severe myocardial injury.

Despite improvement in treatment strategies for STEMI, the incidence of heart failure remains high,^23^ which calls for exploration of new potential markers of early LV function decline. In the early phase of STEMI, previously viable tissue is affected, which is depicted by extensive cardiomyocyte disorganisation, consequently leading to changes in contractile function.^2^ Post-STEMI LV remodelling continues for weeks to several months,^24^ involves both infarcted and remote myocardium^25^ and includes hypertrophy, fibrosis, and inflammation.^26^ Previous studies with first STEMI and comparable infarct size to our study^2,15^ showed that one-third of patients developed adverse LV remodelling, which was defined as an increase in LVEDV_indexed_ >20% at 12 months follow-up^2^ or as an increase in LVESV >15% at 4 months follow-up,^15^ respectively. In turn, adverse LV remodelling is associated with worse outcome, including cardiovascular death, hospitalisation for heart failure, and ventricular arrhythmias.^16^ In the present study, heart failure hospitalisation was reported in 11% of patients, which is in line with previous STEMI studies with comparable study population.^27,28^ Although adverse LV remodelling is associated with poor clinical outcome, the presence of adverse LV remodelling itself did not provide additional prognostic information to baseline LV EF, LVEDV, and infarct size.^16^ Exploring alternative measures of LV function on baseline CMR, including HDF, may help to timely identify patients at risk for worse prognosis after STEMI.

LV HDF has been previously studied, both invasively and non-invasively. Experimental animal studies using cardiac catheterisation reported lost or even reversed LV-IVPGs in ischaemic^29^ and heart failure models.^30^ However, due to its invasive nature, this strategy has never entered clinical practice for this purpose. LV-IVPGs have also been assessed non-invasively with transthoracic echocardiography colour Doppler M-mode^31,32^ and speckle tracking,^33^ and with CMR 4D flow imaging. Of these non-invasive techniques, CMR 4D flow imaging has shown to correlate to invasive IVPG measurements^34^ and is currently the gold standard for the assessment of LV HDF.^35,36^ Unfortunately, the clinical applicability for both echocardiography and CMR 4D flow imaging has been limited, mainly due to time-consuming post-processing and limited availability. The present study shows a new LV HDF analysis method that can be performed on routinely obtained CMR long-axis cine images, omitting the need for additional acquisition sequences. Importantly, this method is validated against CMR 4D flow imaging^35^ and post-processing time is substantially shorter than for echocardiography and CMR 4D flow imaging.

Recently, LV-IVPGs on routinely obtained CMR cine images have been explored in pre-capillary pulmonary hypertension,^37^ dilated cardiomyopathy,^19^ and in heart failure with preserved LV EF,^14,34^ showing its potential in the detection of subtle LV function decline. Also in STEMI, estimation of LV-IVPGs is gaining ground. A recently published study in patients with first STEMI showed comparable LV-IVPGs during cardiac cycle^15^ to our study. Importantly, this study also included 21 non-athletic healthy individuals as a control group. As expected, compared with our STEMI patients, the amplitude of both systolic and diastolic LV-IVPGs appears to be higher in their controls.^15^ In addition, in a cohort of patients with subsequent adverse LV remodelling, significantly lower systolic lateral–septal LV-IVPGs and higher diastolic lateral–septal/longitudinal force ratio were reported, whereas LV EF and LV GLS did not significantly differ.^15^ However, the association between LV-IVPGs and clinical outcome has not been investigated. In the present study, we showed that overall LV-IVPGs were significantly associated with MACE when adjusted for commonly used determinants for adverse LV remodelling, including measures of myocardial injury and clinical covariates. Regarding LV function, LV-IVPGs, LV EF, and LV GLS were all univariably associated with MACE, but the significant association between LV-IVPG and MACE did not hold after adjustment for LV EF and LV GLS. This may partly be explained by the observation that the patients suffering MACE had more pronounced systolic dysfunction in the early phase after STEMI, as demonstrated by worse LV EF, LV GLS, and systolic LV-IVPGs, whilst diastolic LV-IVPGs were still preserved. In addition, reduction in LV-IVPG amplitude significantly correlated with worsening of LV EF and LV GLS. Importantly, LV GLS already showed incremental prognostic value to conventional LV function imaging parameters in STEMI patients.^38–40^ It may be that HDF analysis excels in the detection of subtle diastolic dysfunction, as was observed in pre-capillary pulmonary hypertension,^37^ but that the early phase after STEMI is dominated by systolic alterations, which appears to be better represented by deformation imaging analysis.

In addition to LV function analysis, CMR allows for tissue characterisation and assessment of infarct severity. In this study, we assessed infarct severity by the infarct size, infarct location, and the presence of MVI. Nowadays, MVI is a well-known marker of severe myocardial injury.^41,42^ MVI is associated with worse LV function,^43^ and adverse clinical outcomes, including heart failure hospitalisation and cardiac death,^44,45^ as also observed in the present study. Here, we demonstrated that patients with severe myocardial injury show smaller amplitudes of all systolic and diastolic LV-IVPGs compared with patients with smaller infarcts or infarcts without MVI. Additionally, patients with severe myocardial injury have a higher lateral–septal/longitudinal force ratio, which implies that the resultant LV-IVPG is more orthogonal to the main flow direction and thus less effective in moving blood in the apex–base direction. The overall LV-IVPG remained significantly associated with MACE when adjusted for infarct size and the presence of MVI, suggesting that the impaired LV-IVPGs in these patients could partly explain the observation of worse clinical outcome.

### Clinical relevance

CMR-derived LV-IVPG assessment can provide insight into HDF alterations post-STEMI. The easy application of post-hoc LV-IVPG estimation in readily available data (i.e. routinely obtained steady-state free precession long-axis cine imaging) supports the implementation in daily clinical practice. Moreover, inter- and intraobserver variability of LV-IVPG assessment is limited.^19,46^ A major advantage of LV-IVPG analysis is that it can detect subtle alterations in the entire cardiac cycle rather than merely systolic changes as assessed by LV EF and LV GLS. However, the added value of LV-IVPG analysis in the early phases post-STEMI in predicting MACE seems limited.

### Limitations

This is the first study that assesses the association between CMR-derived LV HDF and prognosis after STEMI. HDF analysis is based on several estimations and calculations. First, it reconstructs a 3D LV using ECG-gated alignment of the endocardial contours in long-axis cine images. Secondly, it estimates the blood volume that passes the valves using the aortic and mitral valve orifice areas. Consequently, HDF analysis assumes sufficient ECG-gating and no valvular pathology. As we excluded patients with atrial fibrillation and severe valvular pathology, the generalisability of our findings is somewhat reduced. Direct comparison of this method to invasive measurements is not (yet) available, but it is validated against the current gold standard CMR 4D flow.^35^

## Conclusion

This study shows that CMR-derived LV-IVPGs are associated with cardiovascular death and *de novo* heart failure hospitalisation when adjusted for common clinical risk factors and measures for myocardial injury. However, in this cohort, LV-IVPG does not add prognostic value in addition to LV EF and LV GLS.

## Supplementary Material

qyae009_Supplementary_Data

## Data Availability

The data underlying this article are available in the article and in its online Supplementary material.

## Lead author biography

Robin Nijveldt is a Professor of Cardiovascular Imaging and Cardiologist at the Radboud University Medical Center (Nijmegen, The Netherlands), and connected to the Radboud Institute for Health Sciences. He works at the Department of Cardiology and focuses on the vascular damage research theme, with a special interest in Cardiovascular Magnetic Resonance imaging, CT, and echocardiography, and in close collaboration with the Department of Radiology. He explores new imaging techniques and innovative strategies on today’s scientific health challenges and aims to improve patient outcome in a personalised approach.
