# Supplementary material for: Cardiovascular magnetic resonance imaging-derived intraventricular pressure gradients in ST-segment elevation myocardial infarction: a long-term follow-up study
Source: Eur Heart J Imaging Methods Pract. 2024 Feb 9;2(1):qyae009. doi: 10.1093/ehjimp/qyae009 (PMC11195698; doi:10.1093/ehjimp/qyae009)
Supplement: qyae009_Supplementary_Data [file qyae009_Supplementary_Data.docx]

| **Supplemental Table 1. Characteristics of STEMI patients with and without event-free survival** | | | |
| --- | --- | --- | --- |
|  | **MACE**  **(*n*=49)** | **No MACE**  **(*n*=258)** | ***p*-value** |
| Age, years | 66.1±10.2 | 56.8±11.1 | <0.001 |
| Male, n (%) | 40 (81.6) | 224 (86.8) | 0.337 |
| Body surface area, m^2^ | 1.9±0.1 | 1.9±0.2 | 0.372 |
| Follow-up duration, years | 8.8 [4.5-12.1] | 9.8 [6.2-13.1] | 0.122 |
|  |  |  |  |
| **Medical history, n (%)** |  |  |  |
| Diabetes mellitus | 8 (16.3) | 38 (14.7) | 0.774 |
| Family history of CAD | 4 (8.2) | 23 (8.9) | 1.000 |
| Hypercholesterolaemia | 14 (28.6) | 98 (38.0) | 0.210 |
| Hypertension | 26 (53.1) | 110 (42.6) | 0.178 |
| Smoking, *current or in the past* | 24 (49.0) | 173 (67.1) | 0.016 |
|  |  |  |  |
| **Coronary angiography** |  |  |  |
| Time to reperfusion, min | 205.0 [158.0-300.0] | 176.0 [133.5-257.0] | 0.029 |
| Multivessel disease, n (%) | 12 (24.5) | 66 (25.6) | 0.872 |
|  |  |  |  |
| **CMR left ventricular parameters** |  |  |  |
| Infarct size (% LVM) | 28.2 [16.8-41.3] | 18.8 [9.9-27.6] | <0.001 |
| MVI, n (%) | 29 (59.2) | 111 (43.0) | 0.037 |
| LV EF (%) | 44.5±11.9 | 51.7±10.5 | <0.001 |
| LV GLS (%) | -10.2±5.0 | -13.6±4.4 | <0.001 |
| Overall LV-IVPG (*i.e.,* longitudinal force) | 9.7±3.4 | 10.9±3.7 | 0.028 |
| Lateral-septal force | 2.6±1.0 | 2.8±0.92 | 0.318 |
| Ratio lateral-septal/longitudinal force (%) | 28.0±7.5 | 26.4±7.3 | 0.165 |
| Systolic ejection ‘A’ | 16.2±7.2 | 18.5±7.3 | 0.043 |
| Peak systolic force ‘A-peak’ | 25.6±11.1 | 29.2±11.5 | 0.048 |
| Systolic-diastolic transition ‘B’ | -6.0±2.5 | -6.4±2.4 | 0.352 |
| Systolic slowdown ‘B1’ | -5.0±3.3 | -5.2±2.6 | 0.733 |
| Diastolic suction ‘B2’ | -6.9±3.0 | -7.2±3.5 | 0.525 |
| B-wave reversal, *n* % | 20 (40.8) | 64 (24.8) | 0.021 |
| E-wave deceleration ‘C’ | 6.0±2.8 | 6.8±3.3 | 0.083 |
| A-wave acceleration ‘D’ | -3.6±2.4 | -3.9±2.1 | 0.477 |
| Values are presented as mean ± standard deviation, median [IQR] or absolute number (%) and assessed by independent-samples T test, Mann-Whitney U test or Chi-square test, respectively. CAD, coronary artery disease; CMR, cardiac magnetic resonance imaging; EF, ejection fraction; GLS, global longitudinal strain; IVPG, intraventricular pressure gradient; LV, left ventricle; LVM, left ventricular myocardial mass; MVI, microvascular injury. | | | |


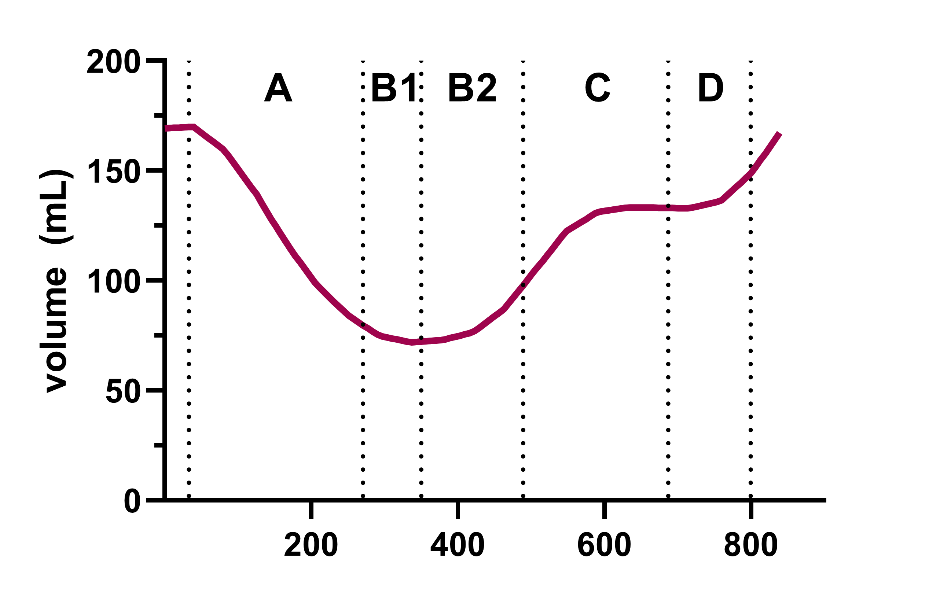

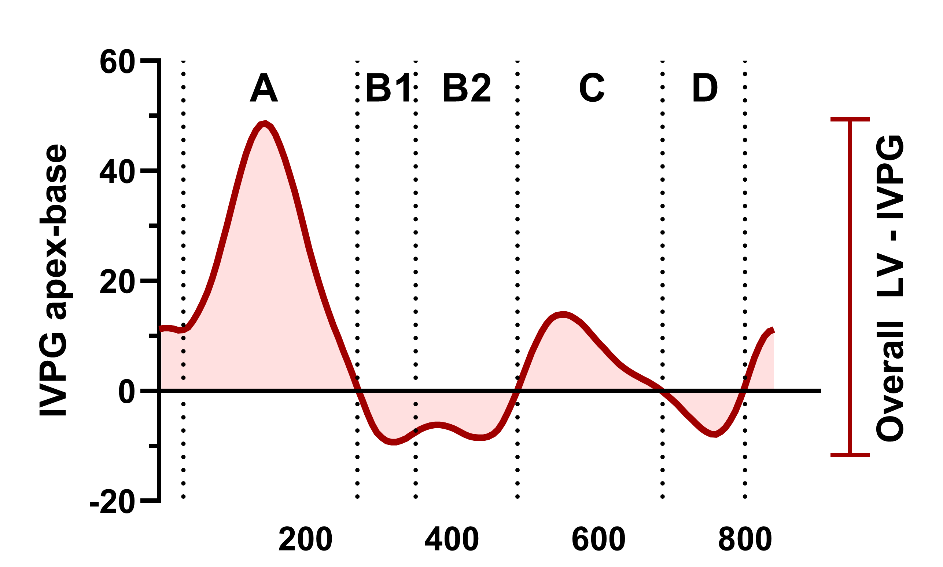


**Supplemental Fig 1 Example of apex-to-base LV-IVPG time curve analysis.** Demonstration of the apex-to-base LV-IVPGs (dimensionless, y-axis) during one cardiac cycle (in ms, x-axis), with corresponding volume curve below (in mL on y-axis and ms on x-axis). In this time curve, five distinct phases can be distinguished. First, the positive vector ‘A’ represents the systolic ejection phase. Second, the negative vector ‘B’ represents the systolic-diastolic transition. ‘B’ consists of the end-systolic LV contraction slow down phase and aortic valve closure ‘B1’, followed by the opening of the mitral valve and diastolic suction ‘B2’. Fourth, the positive vector ‘C’ represents the passive filling phase. Fifth, the negative vector ‘D’ represents the left atrial contraction in late diastole. The area under the curve represents the overall apex-to-base LV-IVPG. LV-IVPG, left ventricular intraventricular pressure gradients.


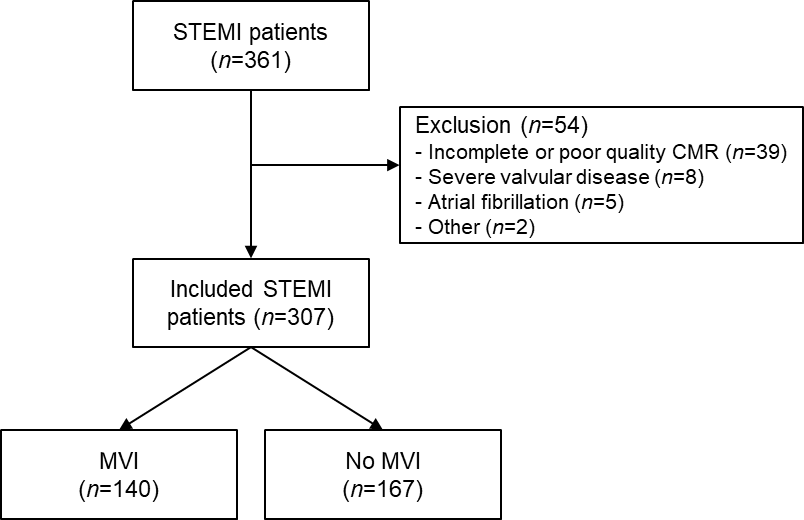


**Supplemental Fig 2 Flow chart of study inclusion.** In total, 307 STEMI patients were included, of whom 140 showed microvascular injury on CMR. CMR, cardiac magnetic resonance imaging; STEMI, ST-elevation myocardial infarction; MVI, microvascular injury

A

B


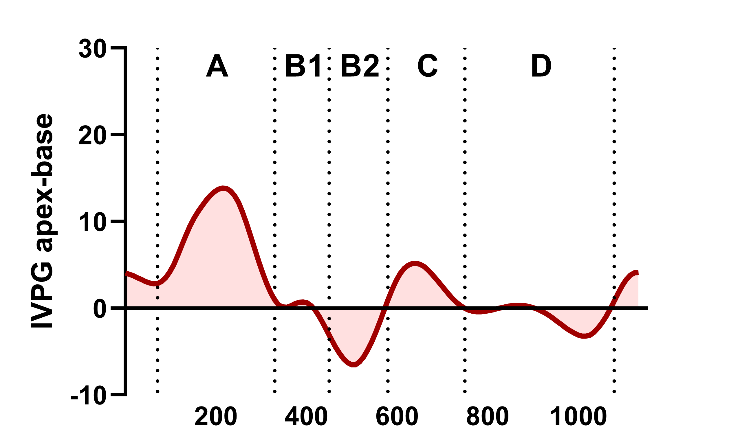

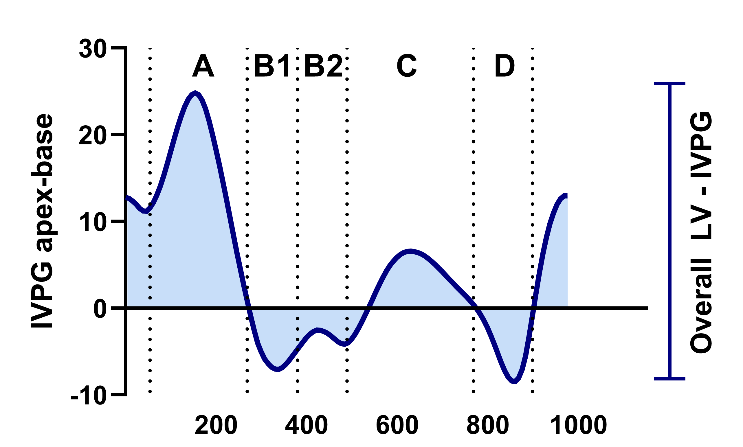


**Supplemental Fig 3 Example of apex-to-base LV-IVPG analysis in a patient with (A) and without (B) microvascular injury.** Patients with microvascular injury showed a significantly lower overall apex-to-base LV-IVPG amplitude (area under the curve) and correspondingly impaired systolic ejection ‘A’, systolic-diastolic transition ‘B’, passive filling ‘C’ and atrial contraction ‘D’. The y-axis represents the LV-IVPG from apex-to-base. The x-axis represents the time in milliseconds during one cardiac cycle. LV-IVPG, left ventricular intraventricular pressure gradients.
